# Mammary cancer initiation and progression studied with magnetic resonance imaging

**DOI:** 10.1186/s13058-014-0495-6

**Published:** 2014-12-16

**Authors:** Xiaobing Fan, Devkumar Mustafi, Erica Markiewicz, Marta Zamora, James Vosicky, Abby Leinroth, Jeffrey Mueller, Kay Macleod, Suzanne D Conzen, Gregory S Karczmar

**Affiliations:** 1 Department of Radiology, MC2026, The University of Chicago, 5841 S. Maryland Avenue, Chicago, 60637 IL USA; 2 Department of Pathology, MC6101, The University of Chicago, 5841 S. Maryland Avenue, Chicago, 60637 IL USA; 3 Ben May Department for Cancer Research, GCIS W421, The University of Chicago, 929 East 57th Street, Chicago, 60637 IL USA; 4 Medicine, Hematology/Oncology, SBRI J301 MC 2115, The University of Chicago, 5841 S. Maryland Avenue, Chicago, 60637 IL USA

## Abstract

**Introduction:**

Previous work from this laboratory demonstrated that magnetic resonance imaging (MRI) detects early murine mammary cancers and reliably differentiates between *in situ* and invasive cancer. Based on this previous work, we used MRI to study initiation and progression of murine mammary cancer, and monitor the transition from the *in situ* to the invasive phase.

**Methods:**

In total, seven female C3(1) SV40 Tag mice were imaged every two weeks between the ages of 8 to 23 weeks. Lesions were identified on T2-weighted images acquired at 9.4 Tesla based on their morphology and growth rates. Lesions were traced manually on MR images of each slice. Volume of each lesion was calculated by adding measurements from individual slices. Plots of lesion volume versus time were analyzed to obtain the specific growth rate (SGR). The time at which *in situ* cancers (referred to as ‘mammary intraepithelial neoplasia (MIN)’) and invasive cancers were first detected; and the time at which *in situ* cancers became invasive were recorded.

**Results:**

A total of 121 cancers (14 to 25 per mouse) were identified in seven mice. On average the MIN lesions and invasive cancers were first detected when mice were 13 and 18 weeks old, respectively. The average SGR was 0.47 ± 0.18 week^-1^ and there were no differences (*P* >0.05) between mice. 74 lesions had significantly different tumor growth rates before and after ~17 weeks of age; with average doubling times (DT) of 1.88 and 1.27 weeks, respectively. The average DT was significantly shorter (*P* <0.0001) after 17 weeks of age. However, the DT for some cancers was longer after 17 weeks of age, and about 10% of the cancers detected did not progress to the invasive stage.

**Conclusions:**

A wide range of growth rates were observed in SV40 mammary cancers. Most cancers transitioned to a more aggressive phenotype at approximately 17 weeks of age, but some cancers became less aggressive. The results suggest that the biology of mammary cancers is extremely heterogeneous. This work is a first step towards use of MRI to improve understanding of factors that control and/or signal the development of aggressive breast cancer.

**Electronic supplementary material:**

The online version of this article (doi:10.1186/s13058-014-0495-6) contains supplementary material, which is available to authorized users.

## Introduction

The processes that characterize and trigger progression of pre-invasive ductal carcinoma *in situ* to invasive ductal carcinoma breast cancer are not well understood [1]. This process cannot ethically be studied in patients. There is therefore a critical need for non-invasive studies of breast cancer progression in animal models. Here we report on serial magnetic resonance imaging (MRI) studies of growth patterns of *in situ* and invasive mammary cancer in a murine model of breast cancer.

Transgenic mouse models of human breast cancer provide an excellent experimental framework for studying the natural history of *in situ* and invasive cancer [2]–[5]. The female C3(1) SV40 Tag transgenic mouse is a widely used model for breast cancer research [6]–[9]. SV40 Tag mice develop mammary cancer that resembles human ductal breast carcinoma, including progression through atypical ductal hyperplasia (∼8 weeks of age), *in situ* cancers (referred to as mammary intraepithelial neoplasia (MIN) in mice) (∼12 weeks of age) and invasive ductal carcinoma (∼16 weeks of age) [10],[11]. MIN is similar to human ductal carcinoma *in situ* and is considered a precursor to invasive cancer [12]. Due to the small size of *in situ* mammary neoplasias in mouse models, high-resolution imaging techniques are required to study how lesions develop, grow and progress over time.

Previous work in this laboratory demonstrated that non-invasive T2-weighted MRI can reliably detect small *in situ* cancers and distinguish between *in situ* and invasive cancers [2]. Jansen and colleagues in this laboratory studied SV40 Tag mouse mammary cancer progression from 10 to 21 weeks of age using MRI [3]. Their research produced image-based evidence that some *in situ* mammary cancers do not progress to invasive cancers. However, these previous imaging studies were limited to only one side of mouse inguinal glands and images were acquired using spoiled gradient echo imaging at 2-week or 3-week intervals between 10 and 21 weeks of age. Generally, more cancers develop in the upper mouse mammary gland than in the lower mammary gland [5]. Therefore, whole mouse imaging that allows evaluation of all of the mammary glands is necessary in order to gain a better understanding of mammary cancer progression.

In this research, we used whole-body MRI screening for lesion assessment in all SV40 mice mammary glands from ~8 to ~23 weeks of age, at 2-week intervals. Mammary cancers were identified and characterized on high-resolution T2-weighted images to follow the transition from *in situ* to invasive cancer. The volumes of lesions were measured and analyzed to determine the specific growth rate (SGR).

## Methods

### Animals

Female C3(1) SV40 Tag transgenic mice (*n* = 7) were studied. All procedures were carried out with approval from the University of Chicago’s Animal Care and Use Committee. Mice were imaged approximately every 2 weeks beginning at age ~8 weeks and ending at ~23 weeks. Animals were anesthetized prior to imaging experiments, and anesthesia was maintained during imaging as 1.5% isoflurane. Temperature, heart rate and respiration rate were monitored with an optical detection system from SA Instruments (Stony Brook, NY, USA), designed for use in small animal MRI. The temperature was kept within normal range using a temperature-controlled fiber optic probe and heating system. The respiration rate was maintained at ~55 breaths/minute and used to obtain gated images.

### Imaging protocols

MRI experiments were performed on a 9.4 Tesla Bruker (Billerica, MA, USA) small animal scanner with 11.6 cm inner diameter and actively shielded gradient coils (maximum constant gradient strength for all axes: 230 mT/m). After being fitted with physiological monitoring equipment, mice were taped into a plastic semicircular holder and placed inside a quadrature coil (outside diameter/inside diameter = 59/35 mm, length = 38 mm; Bruker). Axial low-resolution gradient echo images (repetition time/echo time = 600/3.2 milliseconds, field of view = 25.6 mm, array size = 128^2^, slice thickness = 1.0 mm, number of slices = 41, flip angle = 30°, number of excitations = 2) with fat suppression and respiratory gating were acquired first to ensure that the area of interest was at the center of the magnet and detector. Axial high-resolution multislice rapid acquisition with relaxation enhancement spin echo T2-weighted images (repetition time/echo time _effective_ = 4,000/20.3 milliseconds, field of view = 25.6 mm, matrix size = 256^2^, slice thickness = 0.5 mm, slice gap = 1 mm, number of slices = 41, number of excitations = 2, rapid acquisition with relaxation enhancement factor = 4) with fat suppression and respiratory gating were then acquired. First a complete set of images were acquired for the upper (thoracic, cervical, axillary) glands, and then a complete set of images were acquired for the lower (inguinal) mammary glands. For both the upper and lower glands, two interleaved sets of images were acquired to cover the slice gaps and then combined together for a total of 82 slices. Thus, a total of 164 T2-weighted slices were acquired for each mouse (82 from the upper glands, and 82 from the lower glands).

### Image analysis

Data analysis was performed using programs written in IDL (Exelis VIS, Inc., Boulder, CO, USA). A researcher (XF) with more than 15 years of animal MRI experience and a veterinary technician (EM) with 8 years of small animal imaging experience analyzed all of the images to identify the tumors. Previous studies of this model correlated features identified on MRI with histology and established that small scattered foci with largest diameter between 100 and 400 μm, intensity 1.5 times that of muscle or greater on T2-weighted images, and with elongated morphology (resembling individual ducts) are almost always *in situ* cancers [3]. Solid lesions larger than 500 μm in largest diameter are always invasive cancer. Lymph nodes were identified based on their location, oval shape and signal intensity close to that of muscle. In addition, lymph ducts were identified based on their chain-like morphology and their direct connection to lymph nodes. This morphology clearly distinguished lymph ducts from *in situ* cancers.

For each cancer identified, we recorded the age (of the mouse) at which MIN lesions were first detected (T_0_(MIN), weeks) and the age at which they were last detected (T_1_(MIN), weeks), generally due to the transition to the invasive phenotype, and the age at which invasive cancers (T_0_(IC), weeks) were first detected. The total time (D_MIN_, weeks) over which each MIN lesion was detected was then calculated as follows:

1 $$D_{MIN}=T_{1}\left(MIN\right)-T_{0}\left(MIN\right)+1\text{.}$$

Thus, if a MIN lesion was detected only once (that is, T_1_(MIN) = T_0_(MIN)), then D_MIN_ is equal to 1 week.

The area of each tumor was manually traced on each slice in which the tumor appeared on T2-weighted magnetic resonance images, and multiplied by the slice thickness to determine the volume of the tumor in each slice. All of the slice volumes for each tumor were then added together to estimate the final tumor volume [13]. The tumor SGR (ρ) was calculated by fitting the tumor volumes as a function of time (*V*(*t*)) to the following equation [14]:

2 $$V_{\mathrm{fit}}\left(t\right)=V_{0}\exp\left(\rho \left(t-t_{0}\right)\right)\text{,}$$

where *t* (weeks) is the age of the mouse and *V*_0_ is tumor volume detected at initial time *t*_0_ (weeks). To evaluate the goodness of fit, an average value was calculated for each curve:

$$r=\left|V\left(t\right)-V_{\mathrm{fit}}\left(t\right)\right|/\left(V\left(t\right)+V_{\mathrm{fit}}\left(t\right)\right)$$

If *r*_ave_ <0.05, the fit was considered excellent.

In many cases the growth curves could not be well approximated (*r*_ave_ > 0.05) by the simple mono-exponential model (Equation 2), and two different stages of growth were apparent. In those cases, we identified a transition time (TT) at which the growth rate changed, and determined the doubling time (DT) before and after the TT. The DT, rather than the SGR, was used for this comparison because of the small number of time points available before and after the TT. The comparison of the DT assumes that tumor volumes satisfy Equation 2 before and after the TT with two different growth rates. To estimate the TT, we defined:

3 $$r\left(t\right)=\left(V\left(t\right)-V_{\mathrm{fit}}\left(t\right)\right)/\left(V\left(t\right)+V_{\mathrm{fit}}\left(t\right)\right)\text{,}$$

for each cancer. If the value of *V*_fit_(*t*) is close to the experimentally measured volume *V*(*t*), then *r* is very close to 0. If *V*_fit_(*t*) is significantly less than or greater than the measured volume, then *r* approaches –1 or 1. We applied two-means clustering to *r*(*t*) to identify two clusters around values of *r* close to zero and values of *r* different from zero; these are designated clusters ‘0’ or ‘1’. For each cluster, composed of *r*(*t*) values measured at different ages, the maximum age in each cluster was determined. The TT was determined as the minimum of these two maximum ages. This approximately corresponds to the age that separates the two clusters. This method was only applied to cancers that were sampled at a minimum of four time points, and for which the determined TT was not the first or last time point.

For tumors with two different growth rates, the DT before and after the TT was calculated from the following formula [14]:

4 $$DT=\left(t_{2}-t_{1}\right)\ln2/\ln\left(V_{2}/V_{1}\right)\text{,}$$

where *t*_1_ and *t*_2_ are two different ages, and *V*_1_ and *V*_2_ are the tumor volumes at these two ages. For time points before the TT, *t*_2_ is the age at the TT and *t*_1_ is the age at the scan immediately before the TT. The DT is therefore the growth rate immediately before the week at which the transition occurs. Similarly, for time points after the TT, *t*_1_ is the age at the TT and *t*_2_ is the scan immediately after the week at which the transition occurs. The DT therefore gives the growth rate immediately after the TT.

### Statistical analysis

One-way analysis of variance and Tukey's honestly significant difference test were performed to examine whether SGRs were significantly different between seven mice. Paired *t* tests were used to determine whether the DTs before and after the TT were different for pooled data from all seven mice. *P* <0.05 was considered significant.

## Results

**Figure 1:**
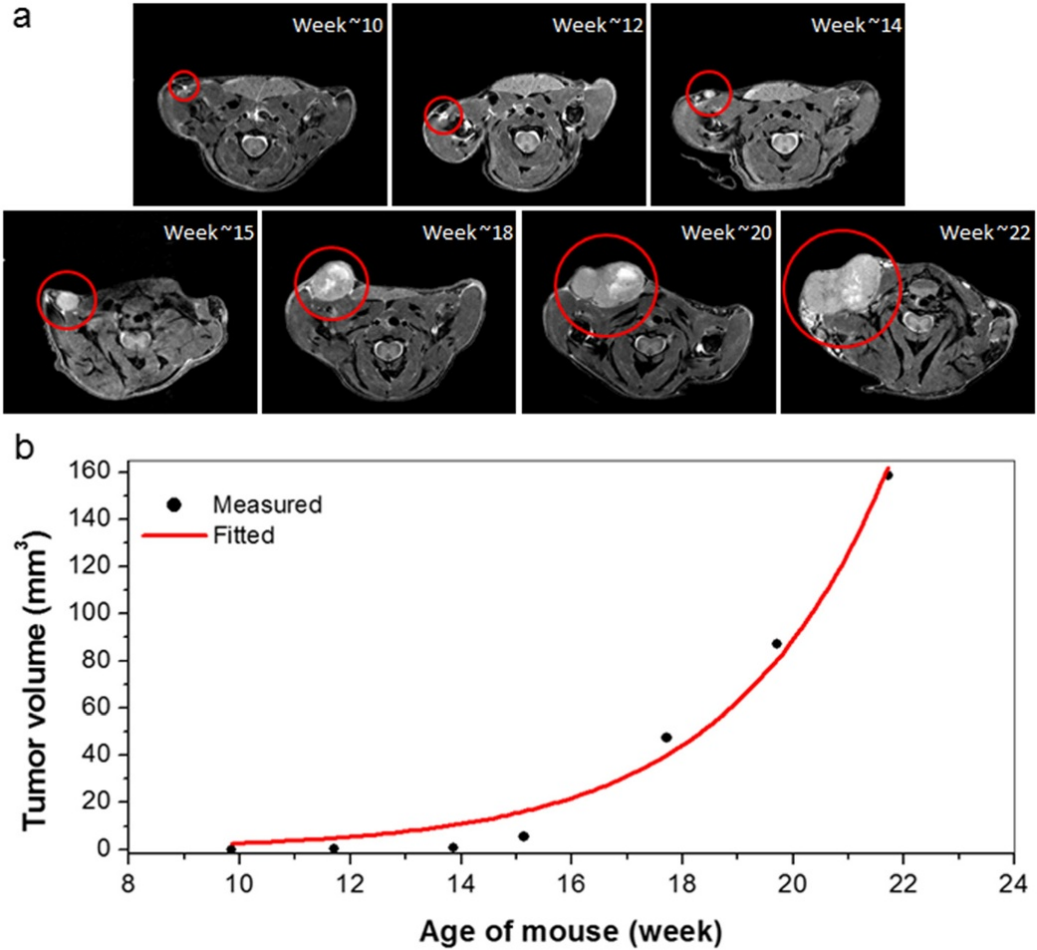
**An identified cancer and corresponding plot of measured tumor volume as a function of mouse age. (a)** A cancer (circled) in the thoracic gland from week ~10 to ~22. A tiny cancer found at week 10 developed into an invasive cancer at week 14, and the size of cancer increased dramatically after that. The displayed image field of view is 25 mm × 20 mm. **(b)** Plot of the tumor volume (black dots) calculated as a function of mouse age and fitted (red line) with the simple exponential tumor growth model.

High-resolution whole body magnetic resonance images successfully detected early *in situ* cancer in all mammary glands. Figure 1a shows a cancer in the thoracic mammary gland from week ~10 to ~22. The cancer is less than 150 μm at largest diameter and its intensity relative to muscle is 1.5:1 at week 10; therefore it is classified as *in situ* cancer. By week 14 the largest diameter of the cancer is 1 mm and it is classified as invasive. After week 14, the size of the cancer increased dramatically. The plot of the volume of this cancer as a function of mouse age is shown in Figure 1b. Equation 2 (red line) does not fit all of the experimental data (dots) accurately, especially at earlier times. It is clear from visual inspection of the plot that there are two different tumor growth rates before and after week ~15.

**Figure 2:**
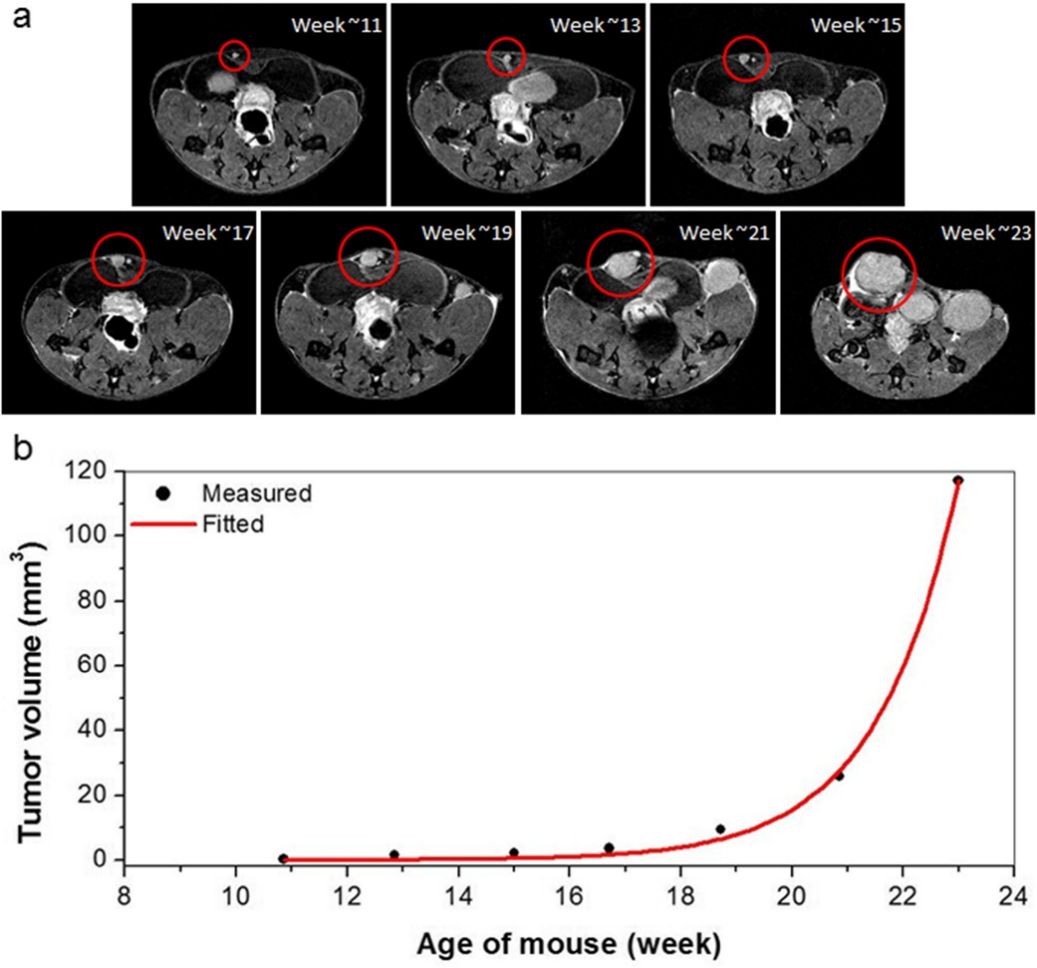
**An identified cancer and corresponding plot of measured tumor volume as a function of mouse age. (a)** A cancer (circled) in the inguinal gland from week ~11 to ~23. Again, a tiny cancer found at week 10 developed into an invasive cancer at week 12, and then the size of tumor increased exponentially after that. The displayed image field of view is 25 mm × 20 mm. **(b)** Plot of the tumor volume (black dots) calculated as a function of mouse age and fitted (red line) with the simple exponential tumor growth model.

Figure 2a shows another cancer in an inguinal gland from week ~11 to week ~23. The cancer is 200 μm at largest diameter with contrast relative to muscle of 1.6:1 at week ~11, and therefore is classified as *in situ* cancer. It develops into an invasive cancer at week ~15, and continues to grow after that. The plot of the volume of this cancer as a function of mouse age is shown in Figure 2b. Equation 2 (red line) fits the data (dots) closely.

**Figure 3:**
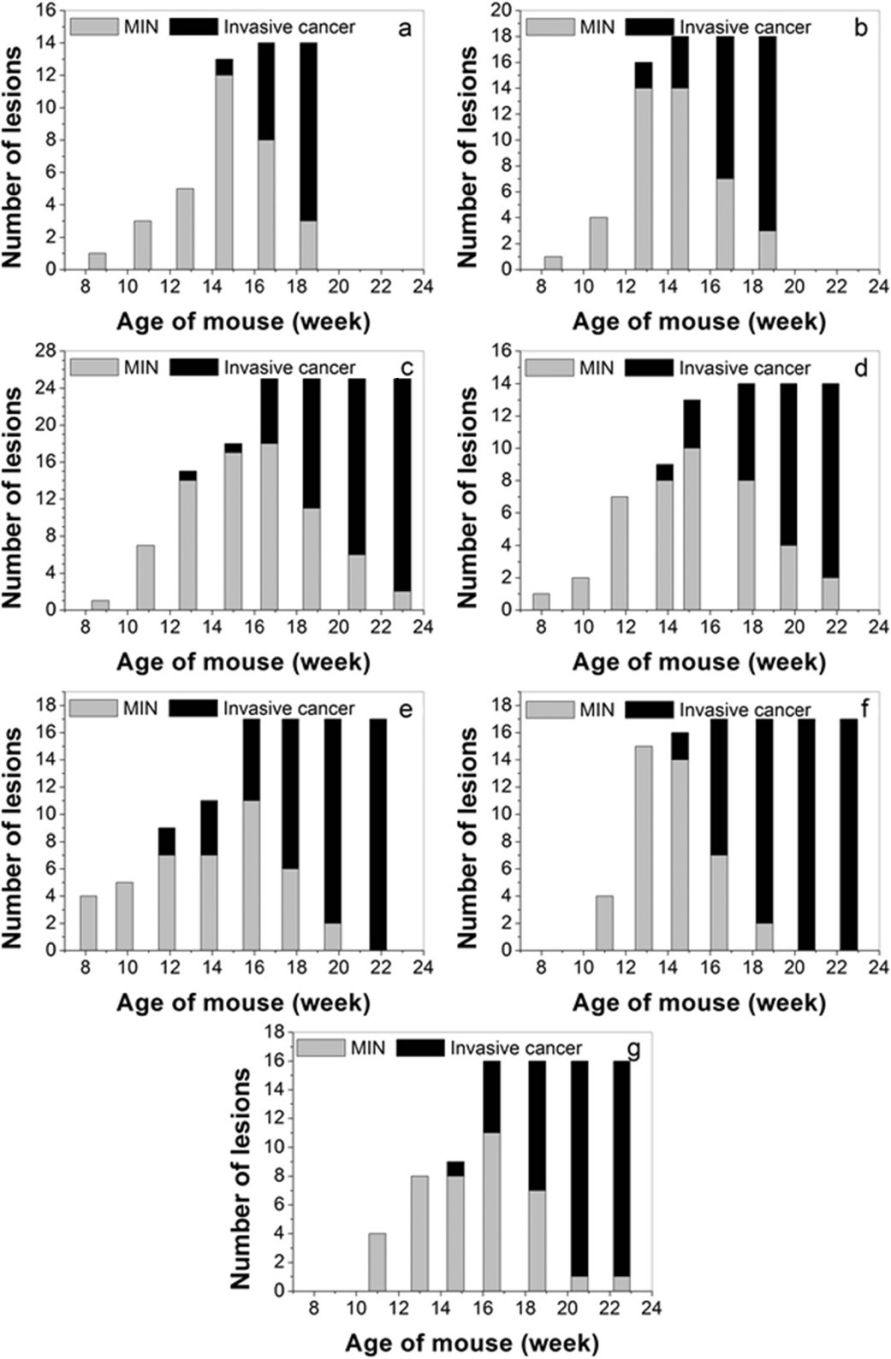
**Number of lesions identified in each scan of each mouse.** **(a)** to **(g)** Stacked bar-plots corresponding with mouse A to mouse G. Total number of lesions found for each mouse ranged from 14 to 25, and more invasive cancers formed after mice were about 18 weeks old. MIN, mammary intraepithelial neoplasia.

The total number of individual lesions found in each mouse ranged from 14 to 25. The numbers of *in situ* and invasive cancers identified in each mouse at each age are shown in Figure 3 (a to g correspond with mouse A to mouse G) in a stacked column plot. For each mouse, Table 1 presents the average age at which each MIN lesion was found, the average age at which each MIN lesion was last detected, the time over which each MIN lesion was detected, and the average age at which each invasive cancer was detected. On average, MIN was first detected at 13.0 weeks, last detected at 16.1 weeks, and the average period over which MIN lesions were detected was 4.1 weeks. Invasive cancer was first detected at an average of 17.6 weeks of age.

**Table 1:** **Age of mice at the time MIN is first detected, average age when MIN is last detected, and average age when invasive cancer is first detected**

Mouse	T _0_(MIN) (weeks)	T _1_(MIN) (weeks)	D _MIN_(weeks)	T _0_(IC) (weeks)
A	13.5 ± 2.1	16.0 ± 1.8	3.5 ± 2.0	17.3 ± 1.3
B	12.5 ± 1.4	15.1 ± 2.4	3.7 ± 1.8	16.5 ± 2.0
C	13.5 ± 2.5	17.6 ± 2.9	5.1 ± 2.3	19.1 ± 2.6
D	13.0 ± 2.6	17.0 ± 3.0	5.0 ± 1.9	16.7 ± 5.0
E	11.9 ± 4.2	14.5 ± 4.8	3.9 ± 2.5	17.3 ± 3.1
F	12.7 ± 1.4	15.5 ± 1.8	3.8 ± 2.0	17.4 ± 1.9
G	14.1 ± 2.3	17.1 ± 2.5	4.0 ± 2.8	18.7 ± 2.2

Data presented as average ± standard deviation. D_MIN_ was calculated using D_MIN_ = T_1_(MIN) − T_0_(MIN) + 1. D_MIN_, total time over which each MIN lesion was detected; MIN, mammary intraepithelial neoplasia; T_0_(IC), average age when invasive cancer is first detected**;** T_0_(MIN), time when MIN is first detected; T_1_(MIN), average age when MIN is last detected.

A small percentage of cancers (10%) did not progress from the *in situ* to the invasive stage during the observation period – on average from ~14 to ~21 weeks. The average (± standard deviation) T_0_(MIN), T_1_(MIN) and D(MIN) for *in situ* cancers that did not progress to the invasive stage was 14.3 ± 2.4 weeks, 20.3 ± 2.0 weeks and 7.0 ± 2.4 weeks, respectively.

**Figure 4:**
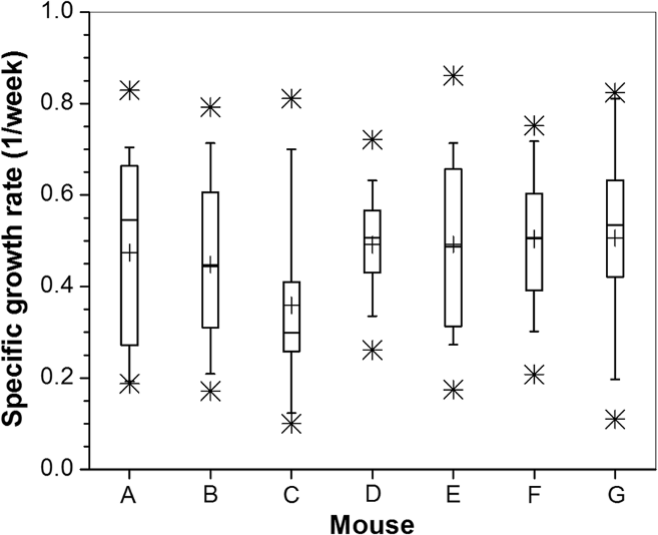
**Specific growth rate for seven different mice.** +, mean; boxes, 25th, 50th, and 75th percentiles; whiskers, 5th and 95th percentiles; *, upper and lower limits of the data.

Figure 4 shows the box plot of SGR calculated by fitting the growth of all lesions in seven different mice to a mono-exponential function. The average SGR was 0.47 ± 0.18/week and analysis of variance showed that there are no significant differences in SGR among the mice.

**Figure 5:**
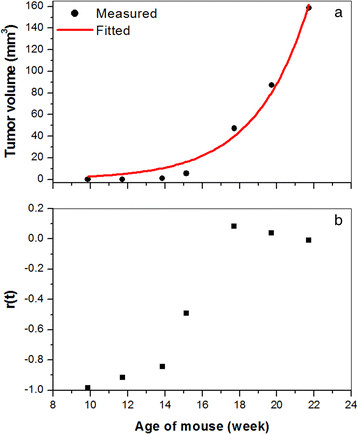
**Determining the transition time from a tumor growth curve. (a)** Plot of the tumor volume (black dots) calculated as a function of mouse age and fitted (red line) with the simple exponential tumor growth model. **(b)** The corresponding plot of *r*(*t*) values as a function of mouse age.

Figure 5 illustrates calculation of the TT for a tumor growth curve that was not accurately fitted by the mono-exponential model. Figure 5a shows the tumor volume and fitted curve as a function of mouse age. Figure 5b shows the corresponding *r*(*t*) values at each time point. Two-means cluster analysis produced cluster ‘0’ containing the four points from 10 to 15 weeks and cluster ‘1’ containing three points from 18 to 22 weeks. Fifteen weeks was identified as the TT for this tumor; that is, the time at which the tumor growth rate changed significantly. In this example, the DT was 0.47 weeks and 0.83 weeks before and after the TT, respectively.

**Figure 6:**
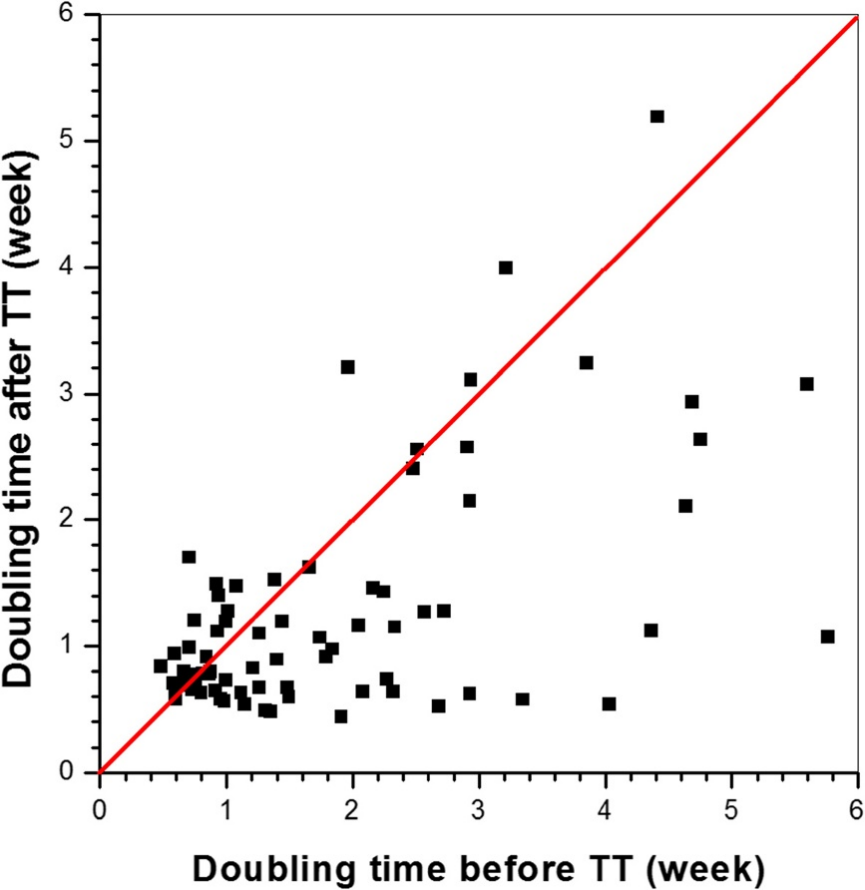
**Doubling time calculated before and after the transition time for 74 lesions.** Red line, the line at which the doubling time after the transition time (TT) = the doubling time before the TT.

Out of 121 cancers identified, the growth curves of 74 cancers were not well approximated by the mono-exponential model and were analyzed as summarized above to obtain two clusters of time points with different DTs. The average TT for these cancers was ~16.6 weeks. This is close to the average time at which invasive cancers were first detected (~17.6 weeks). Figure 6 shows a plot of the DT for 74 cancers before and after the TT. For most cancers (51 of the 74, 69%), the DT was significantly shorter after TT (2.1 vs. 1.1 weeks, *P* <0.000001 by paired *t* test) However, for 31% of cancers the DT was at least 27% longer after the TT; that is, the growth rate of these cancers slowed significantly (an average of 1.3 before TT vs. 1.6 weeks after TT, *P* <0.0001). There was no significant difference in initial volume or volume at 18 weeks between cancers that grew more slowly after the TT and cancers that grew more rapidly after the TT. Nine of the cancers that grew more slowly after the TT and 12 of the cancers that grew more rapidly after the TT were still *in situ* at 18 weeks of age.

**Figure 7:**
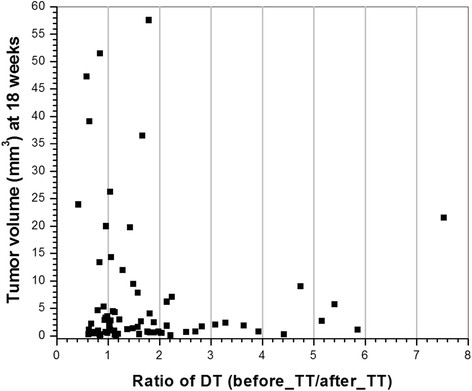
**Tumor volume measured at week 18 versus the ratio of doubling times before the transition time to after the transition time (before/after the transition time), for 74 lesions.** Lesions are the same as those in Figure 6. DT, doubling time; TT, transition time.

Finally, for the 74 lesions shown in Figure 6, we plotted the tumor volume measured at 18 weeks as function of the ratio of the DT before the TT to the DT after the TT (Figure 7). Eighteen weeks of age was chosen because all seven mice were studied at this age. The plot shows that cancers which were larger at 18 weeks tended to exhibit a smaller increase, or a decrease in the DT after TT relative to smaller cancers. The plot emphasizes that while the DT decreased for many cancers after the TT, growth of a significant number of cancers slowed after the TT.

## Discussion

The average ages at which MIN lesions and invasive cancers were first detected, 13 and 18 weeks respectively, were close to those estimated by previous studies of hematoxylin and eosin-stained tissue slices from mice sacrificed at various ages [11]. The majority of MIN lesions identified by MRI progressed to invasive cancers by the age of ~17 weeks. The simple exponential tumor growth model provided poor fits to the data from 74 out of a total of 121 cancers. In these 74 cases there were two different phases of growth with a transition at approximately ~16 weeks of age. This was similar to the average age at which MIN lesions in this group progressed to invasive lesions. For most of the 74 cancers, the DT was significantly shorter after the TT. However, for 31% of cancers, growth slowed significantly after the TT. The cancers that grew more slowly after the TT could not be distinguished from the cancers that grew more rapidly after the TT, based on the initial volume at the time the cancers were first detected, the volume at 18 weeks, or the duration of the *in situ* phase of these cancers.

Growth rates immediately before and after the TT were compared based on the DT rather than the SGR. In principle, the SGR provides a more accurate estimate of tumor growth [15]. However, the goal here was to compare growth rates immediately before and after the TT using only two points. In this case, the DT as calculated here is a more suitable option. Changes in growth rate at 16 weeks suggest a significant change in the biology of the cancers. Since the TT coincides roughly with the average time at which many *in situ* cancers become invasive, this suggests that cancers which successfully escape from the ductal lumens grow much more rapidly when they become invasive. Some cancers may become more indolent at 16 weeks because they are nutrient limited inside the ducts, and are not able to successfully invade and grow outside the ducts.

Previous studies have suggested that *in situ* cancer may be overdiagnosed and overtreated because not all *in situ* cancers will progress to invasive cancer [16]–[20]. The present results show a wide range of progression rates, and a wide range of ages at which MIN and invasive cancers were first detected. A significant number of cancers became more indolent or did not progress during the study period. The fact that this wide range of growth rates are found in a transgenic mouse model that is strongly disposed to develop aggressive cancers is consistent with the view that many human cancers, especially *in situ* cancers, may not be dangerous [21].

The results presented here provide a foundation for the use of the SV40 Tag mouse model (and perhaps other mouse models) to guide the development of improved image-based diagnostic and prognostic parameters that would have important clinical and research applications. In future studies, we will use functional imaging methods (for example, dynamic contrast-enhanced MRI and diffusion-weighted MRI) to identify image-based markers that can reliably predict whether cancers will become aggressive or indolent, and could be used in a clinical setting. Such markers would have an important impact on the clinical management of breast cancer and breast cancer risk. In addition, we will extend the research reported here to obtain more detailed information about the biology, including the genetics, of aggressive and indolent cancer.

By scanning whole body mouse mammary glands, we identified many more cancers than in our previous study, in which only inguinal glands on one side were scanned. This allowed a more meaningful evaluation of the growth patterns of cancers in these mice. Among the 121 cancers identified in all seven mice, more than 60% (77) lesions were identified in the upper glands. In addition, the largest cancer in each mouse often appeared in upper glands. The reasons for this difference between upper and lower glands are not understood, although it may be due to differences in blood supply to the two areas.

Previous work in this laboratory demonstrated that MIN lesions and invasive cancers can be identified and distinguished by MRI based on the signal intensity, size and shape [3]. The methods used here built on this previous research to monitor the transition from *in situ* to invasive cancer. The criteria used to separate *in situ* from invasive cancers are not perfect. However, previous research demonstrated that T2-weighted images have high diagnostic accuracy; using histology as a gold standard. Therefore, it is likely in the present study that there were very few missed or misclassified cancers, and very few false positives.

There are several limitations to this study. First, despite the high diagnostic accuracy mentioned above, some cancers may have been missed due to limited spatial resolution and signal-to-noise ratio. Second, there are inevitable partial volume effects that cause errors in measurements of the tumor volume, especially in the case of smaller *in situ* cancers. Manual delineation of tumor boundaries may have caused additional errors in tumor volume measurements, and computer-aided segmentation may increase accuracy in the future. Finally, mice were scanned every 2 weeks. More frequent samples may be needed, especially at 14 to 18 weeks, to follow the transition from the slow growth phase to the more rapid growth phase.

## Conclusions

Despite these limitations, the present study provides information regarding the growth patterns of *in situ* and invasive murine mammary cancers that has not previously been available. This information provides new insights into the natural history of early murine cancers. Given the similarity between this murine breast cancer model and human breast cancer, these insights are probably also relevant to human breast cancer. The data discussed here as well as future extensions of these experiments can help to address fundamental questions regarding the natural history of ductal carcinoma *in situ*. These questions cannot be answered by studies of humans. Owing to obligate surgical excision of newly diagnosed cancers, lesion progression cannot be followed. Serial imaging of mouse models of breast cancer will therefore be critical for improving understanding of breast cancer, and developing new diagnostic methods and treatments. In particular, we anticipate that the results reported here will lead to the development of functional and anatomic markers that predict the transition from *in situ* to invasive cancer. Such markers would have applications to both routine clinical practice and research.

## Authors’ original submitted files for images

Below are the links to the authors’ original submitted files for images. Authors’ original file for figure 1 Authors’ original file for figure 2 Authors’ original file for figure 3 Authors’ original file for figure 4 Authors’ original file for figure 5 Authors’ original file for figure 6 Authors’ original file for figure 7

## Abbreviations

DT: doubling time
MIN: mammary intraepithelial neoplasia
MRI: magnetic resonance imaging
SGR: specific growth rate
TT: transition time
