# Supplementary figures and images for: Mammary cancer initiation and progression studied with magnetic resonance imaging
Source: Breast Cancer Res. 2014 Dec 16;16:495. doi: 10.1186/s13058-014-0495-6 (PMC4303211; doi:10.1186/s13058-014-0495-6)

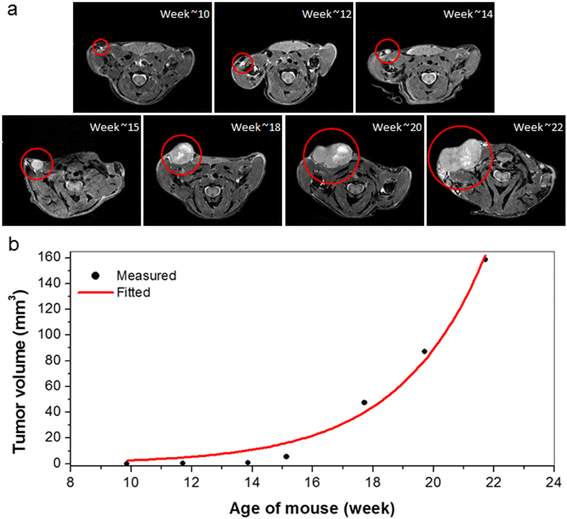

Supplement: Supplementary file 1 — Authors’ original file for figure 1 [file 13058_2014_495_MOESM1_ESM.gif]

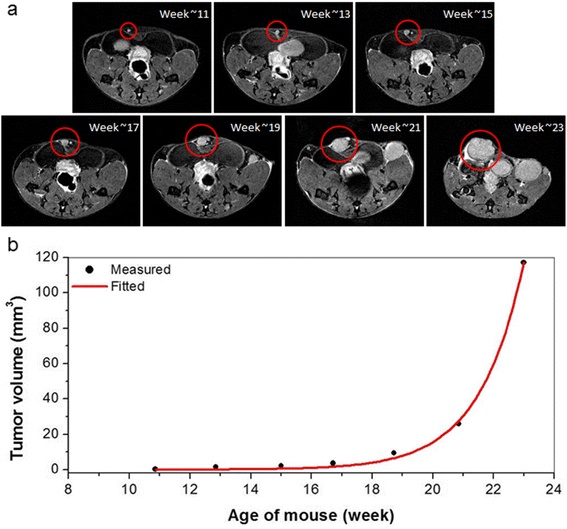

Supplement: Supplementary file 2 — Authors’ original file for figure 2 [file 13058_2014_495_MOESM2_ESM.gif]

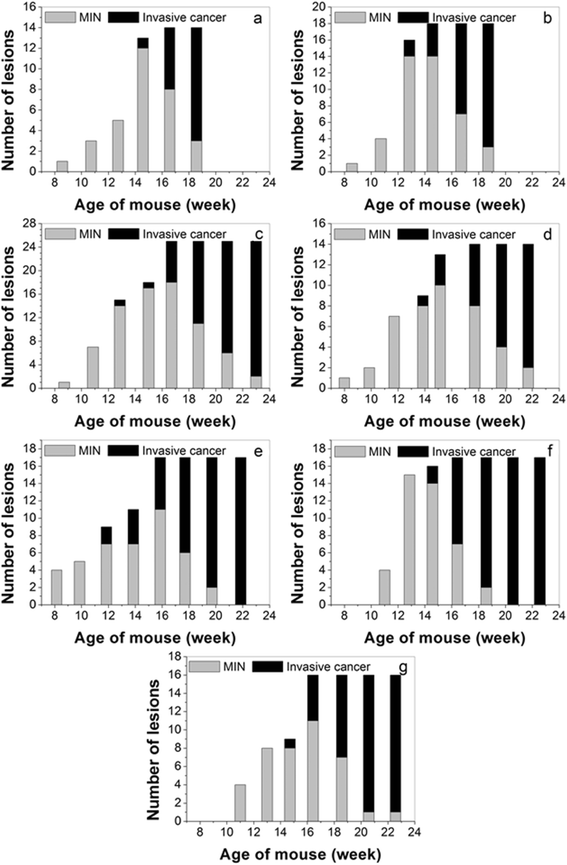

Supplement: Supplementary file 3 — Authors’ original file for figure 3 [file 13058_2014_495_MOESM3_ESM.gif]

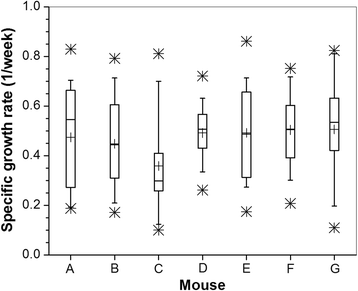

Supplement: Supplementary file 4 — Authors’ original file for figure 4 [file 13058_2014_495_MOESM4_ESM.gif]

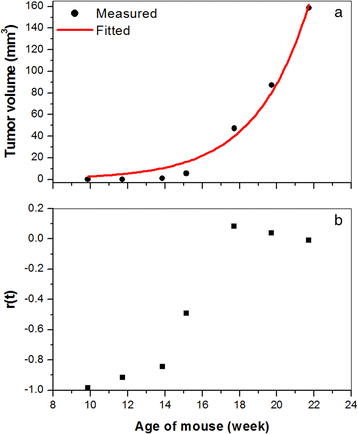

Supplement: Supplementary file 5 — Authors’ original file for figure 5 [file 13058_2014_495_MOESM5_ESM.gif]

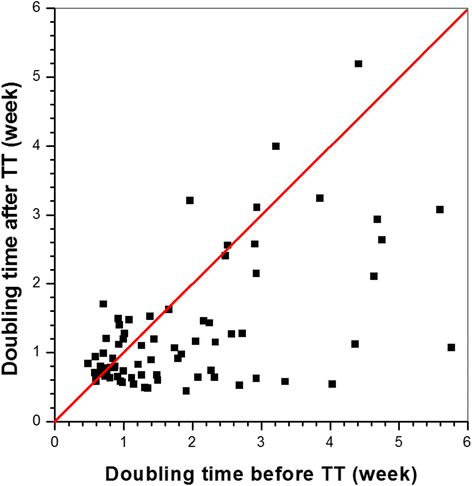

Supplement: Supplementary file 6 — Authors’ original file for figure 6 [file 13058_2014_495_MOESM6_ESM.gif]

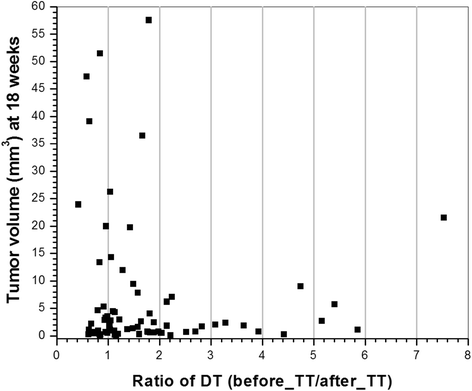

Supplement: Supplementary file 7 — Authors’ original file for figure 7 [file 13058_2014_495_MOESM7_ESM.gif]
